# Optimizing Outpatient Mental Health Services: A REBT-Infused Approach to Empowerment and Well-being

**DOI:** 10.1192/j.eurpsy.2024.1473

**Published:** 2024-08-27

**Authors:** M. Milutinovic, S. Mitrovska, L. Novotni

**Affiliations:** University Clinic of Psychiatry, Skopje, North Macedonia

## Abstract

**Introduction:**

Rational Emotive Behavior Therapy (REBT) fundamentally posits that our thoughts, beliefs, and interpretations exert substantial influence over how we perceive and react to life’s occurrences. Central to REBT is the process of recognizing and disputing irrational, self-defeating beliefs, in favor of adopting rational and constructive perspectives.

**Objectives:**

This presentation endeavors to introduce the foundational principles of REBT, elucidate its applied techniques, demonstrate its efficacy through compelling case studies, and delineate its spheres of applicability.

**Methods:**

Case Studies:

1. Overcoming Social Anxiety: Illustrating the transformation from debilitating social anxiety to enhanced social functioning.
2. Managing Work-related Stress: Exemplifying the alleviation of chronic stress in a high-pressure work environment.
3. Overcoming Depression: Demonstrating the journey from persistent despondency to restored vitality and engagement.

**Results:**

In total, REBT furnishes a methodical and pragmatic approach to therapy, affording individuals agency in steering their emotional well-being towards positive and enduring transformation. It is imperative to acknowledge that the suitability of REBT hinges on the idiosyncratic needs, inclinations, and circumstances of each patient.

**Conclusions:**

By internalizing and applying these foundational principles, REBT empowers individuals to identify and dispute irrational beliefs, paving the way for more adaptive emotional responses and an enhanced overall state of mental well-being. It equips individuals with tangible tools to navigate life’s challenges with heightened resilience and emotional equilibrium.

**Disclosure of Interest:**

None Declared

